# Giving voice

**DOI:** 10.12688/openreseurope.20775.1

**Published:** 2025-07-25

**Authors:** Nenad Ivić

**Affiliations:** 1 University of Zagreb, Faculty of Humanities and Social Sciences, Zagreb, 10000, Croatia

**Keywords:** voice, history, fiction, politics

## Abstract

The expression “giving voice” has replaced the expression “making history” to describe what a historian does. What does it mean? Does this imply a paradigm shift? Has anything changed in the way history is made when it comes to voice? What is voice, and how can its history be written? What role does fiction (or *effet-monde*) play in coming to terms with this elusive concept? Can it point to particular politics of traces shared by both historical and literary writing? Drawing on the works of Giorgio Agamben, Elias Canetti, Michel Foucault, Alain Corbin, and Peter Brown, this article attempts to outline some tentative answers.

                                                                                                                                                                                                                                         Non, tout est une voix et tout est un parfum

                                                                                                                                                                                                                                         Tout dit à l’infini quelque chose à quelqu’un…

                                                                                                                                                                                                                                                  Victor Hugo, *Contemplations*

Today, historiography is about giving voice to the voiceless. Historians used to converse with the dead, today they are called upon to give a voice to those who have been denied it for whatever reason: the illiterate, the oppressed, minorities, and so forth. It is a commonplace. As a succinct expression of the historian’s task, it replaces the expression “making history” ( *faire l’histoire*), especially for those who presumably have no history ( *peuples sans histoire*). Because every person has a kind of history, every person also has a kind of voice. History and voice, the possibility of voice, seem to be universal for human beings: The *homo historicus*, the historical being, and the speaking being, *être parlant*, fundamentally define the human condition. In the second half of the XXth century, we lived in the world of exploded histories; this undoubtedly had to do with the sense of anxiety over one's own destiny that pervaded the world after rapid progress in the post-war period. Today, we live in the world of many voices, no doubt due to technological progress and fear of the Other. At the epistemological level, this change in emphasis is reflected in the subtle shift from culture, scepticism, and language to nature, certainty, and revealed truth. In this respect, when history takes “making” or “giving voice” into consideration, “it simultaneously locates its origins in the actions that ‘produce history’.” ( 1 on page 30). Today, everyone’s voice is readily available as an action that produces the history and demands to be heard and believed. Croce was right: The cacophony of the world we live in makes us long for the cacophony of the worlds of the past. This conscious effort to hear the voices of the voiceless is infused with an unconscious longing to understand the making of the present; we always tend to presentify the past, as if observing history as it unfolds will make it less alien.

Giving voice, like making history, is a metaphorical expression. It connects history with the historian; it summarizes what the historian does, but also gently governs his actions. I will take this connection seriously and try to locate its origin not in the chronological past, but as something that “like a whirlpool in a river, is simultaneous with the becoming of the phenomenon from which it takes its matter and in which it nevertheless remains in a certain way stable and autonomous” ( 2 on page 63). In this sense, the search for its origin means the search for the *ἀρχή*, the origin which is also the mastery of the present. In this case, I will focus on the notion of “voice”. What are we saying when we use the word voice? What senses, meanings, and explanatory powers, apart from banal parroting, are invoked by this seemingly unremarkable blanket term?

Voice, language, and history are connected. “History does not merely touch on language, but takes place in it”, remarks Adorno in *Minima moralia* ( 3 on page 219). “Language is in fact, the result of a construction that is passed on by people through an ultimately historical, esosomatic tradition. On the other hand, this grammaticalized language cannot exist without a voice that embodies it in every speech act, even if it is not entirely clear how this happens”. Furthermore,

human nature is so difficult to define because, being inseparable from language, it takes the form of an articulation between two heterogeneous elements whose relationship is as problematic as the relationship between *φωνή* and *λόγος*. This relationship is not an endosomatic suture acquired as such once and for all, but a historical operation perennially in act. This operation must simultaneously separate and join what is live and what speaks, the human and the non-human; it is enacted incessantly and punctually differed and missed. Anthropogenesis, becoming human of humans, did not take place in some prehistoric archi-past; it is an event that is still taking place and in which philosophy, grammar, logic, psychology, cybernetics and computer science are involved. Man never ceases to become human and to remain inhuman ( 4 on pages 86–88).

As making history, giving voice is nothing less than an element of anthropogenesis. Essentially, those without history are the same as those without voice; history and voice are part of the same historical operation. If we are reasonably well equipped to deal with history and voice as physical phenomenon, we are particularly ill-equipped to deal with voice as a historical phenomenon.

Historians, who like philosophers are indefatigable critics, semanticists, and grammarians, are powerless in the face of voice. As historians, we read texts; very rarely do we pay attention to accents, inflections, scansions, and the prosody of the voice that uttered them. Nevertheless, we want to give a voice to the voiceless. We write them: giving voice means writing voice. For what purpose? Presumably, to produce a dialogue, however flawed or staged it may be. Historians always stage a dialogue, like Thucydides in a dialogue between the Athenians and the Melians. Historian’s dialogue is exclusively about meaning and significance, even if he tries to deconstruct them. Are the traces and hints of voice, gleaned from the sources and transformed into speech, just a kind of modern historian’s *εὔλαλία*, similar to the speeches of Pericles in Thucydides, beautifully crafted in order to confirm his and the historian’s *παιδεία*, aptly translated by Cicero as *humanitas*? When it comes to humanity or inhumanity, “the problem of the voice [becomes] essentially political” ( 4 on page 88). If history is a politics of traces ( 5 on page 193), what can be said about its articulation in the voice when traces of voice are largely unnoticed and irretrievably lost?

In contrast to historiography, at least in the broadest Herodotean sense of inquiry into the past, which has been pursued since the beginning of Western civilization, voice, preservation, and reproduction of voice is a recent phenomenon. History may have become scientific in the XIXth century, but it has always been there. It cannot be compared to the great divide between the voiceless and voiced past ( *cf*. 6 on pages 267–268): unlike the history that can be read, the voice before the invention of the gramophone cannot be heard. It is irretrievably lost. For the historian, a large portion of the past is mute.

Flaubert, for example, subjected his writing to the *épreuve de gueuloir;* he read aloud what he had written, and only if the text passed the test of his vociferation, he was satisfied. We have his text, but we will never know exactly what this ordeal meant. Alternatively, we can learn very much about Louis XIII’s speech habits from the copious notes of his physician, but we cannot hear him ( 7 on pages 484–489). Flaubert and Louis XIII are for us forever silent. The same goes for the Romans, the Greeks, and the medieval Bretons: in spite of all treatises in oratory and remarks of writers such as Pliny the Younger, we do not, and we will never know how Herodotus sounded when he presumably recited his *Histories*, Cicero, when he pleaded his cases, or an anonymous *jongleur* when he recounted *La chanson de Roland* to the audience of soldiers before the battle of Hastings in 1066. It is only recently that we can hear and compare, if we are lucky, Mary Douglas’s voice with Peter Brown’s recollection of her “high whisper, her […] eyes always slightly raised, as if she were reading, from a screen in front of her, the laser-fast succession of her own thoughts” ( 8 on pages 328–329). However, for that, we need a historian like Peter Brown, exceptionally sensitive to the body and the “unexpected sound” ( 8 on page 429). Of course, there is such a thing as oral history, and certain aspects of orality can be reconstructed from silent documents. The study of antique and medieval literature greatly benefited from it. What eludes us still, is not that what “is carried […] by dramatic inflections, subtle stresses, sympathetic accents” that can be gleaned from a text, “but by the *grain* of the voice, which is an erotic mixture of timbre and language” ( 9 on page 66). Barthes lamented the fact that literature severed every link between voice and text and dreamed, with Flaubert on his mind, of a text written aloud. On the other hand, Brown, who also read his texts aloud to himself before offering them to the public, seems convinced that this rendering of the mixture of timbre and language discloses a distinctly human quality of intellectual history, even if it is his own.

We can try to write a history of silence, as Alain Corbin, with an unmistakable flair of gaps in historiography, did in 2016 in his *History of Silence. From renaissance to present day* ^10^. However, what does it mean to write a history of silence about an age that is silent on us? Speaking about rural societies in XIXth century, Corbin notes that “the interplay between silence and speech is extremely complex. The historian must simultaneously distinguish between imposed silences, deliberate silences, implicit silences, instrumentalized silences, and silences resulting from poor command of enunciation, not to mention the refusal of elites to register the peasants’ speech, which was considered poor, awkward and incomprehensible” ( 10 on page 142). This remark aptly summarizes the problem of the opposite: voice. It too can be imposed, deliberate, implicit, instrumentalized, and denied; like Corbin’s silences, the appearance of the sense voiced can be brought to life through the patient work of the historian. However, from our point of view, how can we set all those silences against the silent debris of the past and elucidate the mixture of timbre and language and its eroticism, and write the history of the grain of the voice?

The concept of timbre in speech is a notoriously elusive term. This is part of a repertoire of terms used to define the specificity of voice. Derrida speaks of the “pure differential vibration” as a dream of inaccessible unity and purity “that institutes speech, writing, voice and timbre” ( 11 on pages 144–145), and warns that even in the age of readily available voices, the voice is “totally artificial”. To become available, the voice passes through a kind of “extremely artificial dispositive”. However, the dispositive allows “a certain ‘spontaneity’, to pass through, which shows itself in small remarks about the discomfort of the situation” ( 11 on page 142). Derrida’s remarks on the timbre point to two things: first, the idea of voice is linked to spontaneity, authenticity, and personal experience; and second, if spontaneity and authenticity are unattainable, then, surrounded by a cacophony of voices, we are essentially in the same situation as a historian confronting the silent past and trying to obey the imperative to give voice. His production of the voice of the past must pass through the extremely artificial dispositive of history, thereby shedding its spontaneity and authenticity. It is not the voice that is reconstructed that paradoxically allows spontaneity and authenticity through; it is the remarks about the discomfort of reconstruction that reveal it; they are the personal experience of history in the making.

Postcolonial critics are acutely aware of the problem of the possibility and articulation of voice: “[…] inside or outside the circuit of the epistemic violence of imperialist law and education by supplementing an earlier economic text, can the subaltern speak?” ( 12 on page 269). The problem is not new. Giving a voice to minorities, to women, and to all the subalterns of the world deprived of voice is not different from giving voice to luminaries of ancient culture; in fact, there is no difference between giving voice to a slave or to a labourer and giving voice to Pericles addressing the *ἐκκλησία* of the Athenians. The voice of Greek women is an example of this: women have plenty to say in tragedies; but tragedies are made for men, and the writing of history of Greek women amounts to a “list of limits: map of impossibilities” ( 13 on page 489). We think of Greece and Rome as the cradles of the Western world (and its prejudices); they created for the first time a deliberative space in which a voice could be articulated and heard. For a long time, we have tried to understand them broadly in terms of their origins, whether past or present. With the advent of anthropology of the ancient world, this bond has been somewhat broken. We now endeavour to understand Antiquity in terms of difference and exoticism. Similarly, we tried to understand minorities in terms of our common human origins, tempered by difference and exoticism; this bond has now also been broken, and their own authenticity, seen as a possibility to speak, to have a voice, has taken its place. Through the dispositive of history, they themselves speak articulately. Their painfully reconstructed, authentic articulations are thought to be radically different, and they use the voice of history to demonstrate pain and reconstruction. Precisely because it becomes a historical voice, a voice that articulates itself in history and through the dispositive of history, because it obtemperates to the poetics of history, it loses its authenticity. However, this does not mean that it is imaginary or untrue but only that it is lean and hungry.

If authenticity has any meaning at all in history, there is nothing more inauthentic than the “I have experienced” ( *mon ressenti*: 14 on pages 65–66), the cornerstone of the voice to be given to the other in the staged dialogue of the historian.

I had walked about in the hills where buses did not go. Shy, hardy women gathered leaves and vegetation from the hillside to feed their goats. […] They were the rural subaltern, the real constituency of feminism, accepting their lot as the norm, quite different from the urban female sub-proletarian in crisis and resistance. If I wanted to touch their everyday without the epistemic transcoding of anthropological field work, the effort would be a much greater undoing, indeed, of life's goals, than the effort to catch the Rani in vain, in history. These are the familiar limits of knowing; why do we resist it when deconstruction points at them? ( 12 on page 242)

In depicting her own experience of finding history, Spivak builds her own stage, her own theatre of historical operation: her touching of the everyday of the rural subaltern as a real constituency, that slides effortlessly into the autobiographical cameo of her thwarting of the goal of life, albeit offered in a prudent, conditional mode, provides this scene with certain realisms that are in fact in XXIth century glocalist academisms "that try to reconstitute symbols and offer to the public the occasion to identify itself with the images” of the perennial suffering self ( 15 on pages 122–123). That this suffering self is transmogrified and seems to be duly coached by the historian or the literary critic infected by history in order to audition unsuccessfully for some *Masterchef* of words is another matter. The staging and drama of voice, aiming at a deep consensus, projects itself as *ἀρχή*: the origin and command of “giving voice”, which always, even when framed by Freud’s useful fictions and delivered as a verdict, bypasses the singularity of the grain of the voice.

In *The Voices of Marrakesh*, Elias Canetti, an astute observer of the masses, grapples with his personal experience to examine the grain of the voice:

Here I am, trying to give an account of something, and as soon as I pause I realize that I have not yet said anything at all. A marvellously luminous, viscid substance is left behind in me, defying words. Is it the language I did not understand there, and that must now gradually find its translation in me? There were incidents, images, sounds, the meaning of which is only now emerging; that words neither recorded nor edited; that are beyond words, deeper and more equivocal than words. / A dream: a man who unlearns the world's languages until nowhere on earth does he understand what people are saying. / What is there in language? What does it conceal? What does it rob one of? During the weeks I spent in Morocco I made no attempt to acquire either Arabic or any of the Berber languages. I wanted to lose none of the force of those foreign-sounding cries. I wanted sounds to affect me as much as lay in their power, unmitigated by deficient and artificial knowledge on my part. ( 16 on page 22).

Foucault grappled with the voice of infamous men:

Lives of a few lines or a few pages, nameless misfortunes or adventures gathered into the handful of words. […] The term ‘news’ would fit them rather well, because of the double reference it suggests to the rapid pace of the narrative and to the reality of the events that are related. For the things said in these texts are so compressed that one isn’t sure whether the intensity that sparks through them is due more to the vividness of the words or to the jostling violence of the facts they tell. Singular lives, transformed into strange poems through who knows what twists of fate. […] Here is rarity and not prolixity that makes reality equivalent to fiction. […] There was a kind of immense and omnipresent call for the processing of these disturbances and these petty sufferings into discourse. An unending hum began to be heard, that delivered individual variations of behaviour, shames, and secrets into the grip of power ( 17 on pages 157, 162, 169).

The *Voices of Marrakesh* were published in 1967, *Lives of infamous men* in 1980. Although both are a kind of laminated discourse that seeks to report on something that is named, archived, or travelled, they do not provide verdicts. It is difficult to imagine two more different approaches to giving voice to the other. Canetti’s battlefield is in himself, giving meaning to his own memories of the Other. Foucault’s battlefield is in knowledge, giving historical meaning to documents on marginals, as befits the author of *Die Blendung* on the one hand and the author of *Les Mots et les choses* on the other. However, incidental musings resonate at a deeper level.

Nicole Loraux adds a crucial remark to her list of the impossibilities of women’s history in ancient Greece: “But there remain the flights of free speech which, in Euripides, imitate rather than reproduce the prosaism of everyday language” ^13^. Canetti and Foucault attempt, each in their own way, to come to terms with the marvellously viscid substance of the voice and to understand how it defies words, criticism, semantics, and grammar. Their flights of speech do not degenerate into the “turgid and verbose declamation” ( 18 on page 147) of a theoretically informed historian, nor do they have the “ungainly informativeness of a railway timetable” of a phonetician ( 19 on page 187). They exclude sense and meaning to capture the strange poetic quality of voice’s differential vibrations and the uniqueness of their *tumulte au silence pareil* (their tumult like a deep silence), which constitutes the power of language. The power of the strange-sounding cries that Canetti touches on, and the humming of the infamous men that Foucault delivers into a grip of power, obliquely re-enact rather than reproducing the erotic mixture of timbre and language that constitutes the grain of the voice. They also have their origin and command, but their own voice, which delicately and freely articulates what lies beyond the meaning of language, renders the given meaning fragile ( *cf*. 20 on page 91) and reveals what it conceals, making it more humane, subject to change, and thus more political.

The remarks of Canetti and Foucault are also stations in journeys of the mind, hints, and traces of discomfort in the performance of imaginative re-enactments of the ascendency of voice over meaning. They illustrated what it was like to be in the midst of this power and intensity, to feel it, to let it happen, and to give an account of it. They achieve this, or at least try to, with poetic means, with flights of free language, with what can be called fictive. “What if the fictive was precisely not the beyond nor the intimate secret of the everyday, but the flight of the arrow which hits us right in the eyes and offers us everything which appears. In that case the fictive would also be that which names things, makes them speak and gives them, in language, their being already split by the sovereign power of words” ( 21 on page 308). A witness to a lecture by Peter Brown captures this elusive yet cardinal quality of the fictive concealed in the language that tells someone something: “The stammer came into its own as a rhetorical tool, and he did the holy man in different voices. I still remember the desert father Pambo tempted by demons and carrying feathers on a stick and crying out ‘Go, go’” ( 8 on page 340). Brown spoke to historians and about history; yet he also spoke for the pleasure of speaking ( *λόγου χάριν λέγουσιν,* Aristotle, *Met*. 1011b; *cf*. 22 on page 89) and the holy man, the object of his speech, “became in the eyes of all ‘objectively’ the effect of the omnipotence of the Logos” ( 22 on page 132). Brown’s stammer turned rhetorical tool, “delivered” the poor desert father “from the scorn of Gibbon” ( 8 on page 353): enacting the articulation of his own voice and the historical *logos*, he fleshed out the holy man, and Late Antiquity, as we know it, emerged.

Free speech does not mean an absolute license; it is an act of criticism that transforms the inhuman drabness and muteness of the real into a human, colourful, and noisy reality. Object-orientated and object-free, the flights of free speech in Canetti, Foucault, and Brown plunge us into the vibrant social world of equivocation, polysemy and homonymy, “where we can hear the grain of the throat, the patina of the consonants, the voluptuousness of the vowels, a whole carnal stereophony: the articulation of the body, of the tongue, not that of meaning, of language” ( 9 on pages 66–67) make a difference and thus make sense. Like oil poured on water, their fictive, written voices glisten, floating over tumultuous silence of the past and the deafening cacophony of the present, hitting us in the eye. In their own way, they re-enact the articulation that separates and joins what is live and what speaks, inviting us to observe the perpetual articulation of voice and language for a moment. They make us aware of our own anthropogenesis, of our becoming human. With equal gusto, fictive taps into the exuberant currents of life and the deep corporeal and carnal tides of humanity and, in doing so, risks itself historically. This *Va banque* of the fictive is not to be taken lightly. This reminds us of the precariousness of our own humanity.

## Ethics and consent

Ethical approval and consent were not required.

## Data Availability

No data associated with this article.

## References

[1] de Certeau M : The writing of history. Translated by Tom Conley. New York: Columbia University Press,1988. Reference Source

[2] Agamben G : “Vortici”. In: *Il fuoco e il racconto*. Rome: nottetempo,2014;61–66. Reference Source

[3] Adorno TW : Minima moralia. Reflections on a damaged Life. Translated from the German by E.F.N Jephcott. London: Verso,2005. Reference Source

[4] Agamben G : La voce humana. Macerata: Quodlibet,2023. Reference Source

[5] Boucheron P : Faire profession d'historien. Paris: Seuil,2016. Reference Source

[6] Pomian K : Sur l’histoire. Paris: Gallimard,1999. Reference Source

[7] Rey A Duval F Siouffi G : Mille ans de langue française. Histoire d’une passion. Paris: Perrin,2007. Reference Source

[8] Brown P : Journeys of the mind: a Life in history. Princeton: Princeton University Press,2023. Reference Source

[9] Barthes R : The pleasure of the text. Translated by Richard Miller. New York: Hill and Wang,1998. Reference Source

[10] Corbin A : Histoire du silence. De la Renaissance à nos jours. Paris: Albin Michel,2016. Reference Source

[11] Derrida J : Points de suspension. Entretiens. Choisis et présentés par Elisabeth Weber. Paris: Galilée,1992. Reference Source

[12] Spivak GC : A critique of postcolonial reason. Toward a history of the Vanishing present. Cambridge Massachusetts, London England: Harvard University Press,1999. Reference Source

[13] Loraux N : Notes sur un impossible sujet de l’histoire. In: *La Grèce hors d’elle et autres textes.*Edited by Michèle Cohen-Halimi. Paris: Klincksieck,2021;480–489. Reference Source

[14] Deguy M : L’envergure des comparses. Ecologie et politique. Paris: Hermann,2017. Reference Source

[15] Lyotard JF : Représentation, présentation, imprésentable. In: *L’Inhumain. Causeries sur le temps.*Paris: Klincksieck,2014;117–125.

[16] Canetti E : The voices of Marrakesh. A record of a visit. Translated from the German by J.A. Underwood. New York: The Seabury Press,1978. Reference Source

[17] Foucault M : Lives of infamous men“. In: *Power. Essential works of Michel Foucault*. edited by James D. Faubion, London: Penguin Books,2002;157–175.

[18] Gibbon E : Memoirs of my Life. Edited by Betty Radice. London: Penguin,1990.

[19] Gay P : Style in history. New York: McGraw Hill,1976. Reference Source

[20] Foucault M : About the beginning of the hermeneutics of the self. Lecture at Dartmouth College 1980. edited by Henri-Paul Fruchard et Danielle Lorenzini. Translated by Graham Burchell. Chicago and London: Chicago University Press,2016. Reference Source

[21] Foucault M : Distance, aspect, origine.In: *Michel Foucault. Dits et écrits I 1954-1975*. edited by D. Defert, F. Ewald, J. Lagrange, text no. 17. Paris: Gallimard,2001.

[22] Cassin B : Quand dire, c’est vraiment faire. Homère, Gorgias et le peuple arc-en-ciel. Paris: Fayard,2018. Reference Source

