# Supplementary material for: Giving voice
Source: Open Res Eur. 2025 Jul 25;5:195. [Version 1] doi: 10.12688/openreseurope.20775.1 (PMC12417971; doi:10.12688/openreseurope.20775.1)
Supplement: Peer review history

# Reviewer report: Reviewer response for version 1

**DOI:** 10.21956/openreseurope.22478.r57312

**Published:** 2025-09-08

**Authors:** Matteo Nicolini

**Affiliations:** 1 Verona University, Verona, Italy

The article is well written; the topic is clearly presented, and arguments are finely arranged. I think that the essay does contribute to the cultural, historical, social understanding of how historiography and the 'making of history' should be done in the contemporary era. It correctly addresses the several issues that, for example, sorround the idea of authenticity and that of giving voice to the voiceless. I find particularly compelling the passage on timbre and postcolonial studies.

Is the argument persuasive and supported by appropriate evidence?

Yes

Is the work clearly and cogently presented?

Yes

Is the topic of the essay discussed accurately in the context of the current literature?

Yes

Does the essay contribute to the cultural, historical, social understanding of the field?

Yes

Reviewer Expertise:

Law and humanities, legal traditions, legal (and historical) narratives.

I confirm that I have read this submission and believe that I have an appropriate level of expertise to confirm that it is of an acceptable scientific standard.

# Reviewer report: Reviewer response for version 1

**DOI:** 10.21956/openreseurope.22478.r57307

**Published:** 2025-08-26

**Authors:** A. Arun Daves

**Affiliations:** 1 Jawahar Science College, Neyveli, Tamil Nadu, India

The article, Friendship, Hope and Communication during Second-wave and Postfeminism: Gillian E. Hanscombeʼs Between Friends and Sally Rooneyʼs Conversations with Friends, presents an insightful comparative analysis of two feminist novels within their historical and ideological contexts. The topic is accurately discussed in line with current literature, drawing on influential feminist theorists such as Judith Evans, Rosalind Gill, and José Carregal-Romero. The argument effectively explores how narrative forms—epistolary letters versus fragmented digital communication—mirror the ideological shifts from second-wave feminism’s communal optimism to postfeminist individualism shaped by neoliberalism. While the essay is theoretically rigorous and contributes significantly to cultural and social understanding, its clarity and persuasiveness are partly hindered by dense academic language, heavy citation clusters, and limited direct textual analysis from the novels. Repetition of key ideas without added nuance also weakens coherence. To strengthen the work, the author should integrate close readings and direct quotations from both novels, streamline language for readability, and reduce redundancy while maintaining theoretical depth. Overall, this is an original and valuable contribution that requires revisions to balance theory with textual engagement and enhance clarity for scholarly publication.

Is the argument persuasive and supported by appropriate evidence?

Partly

Is the work clearly and cogently presented?

Partly

Is the topic of the essay discussed accurately in the context of the current literature?

Yes

Does the essay contribute to the cultural, historical, social understanding of the field?

Yes

Reviewer Expertise:

English Literature, Language and literature, Language and Linguistics, American Literature, Commonwealth Literature

I confirm that I have read this submission and believe that I have an appropriate level of expertise to confirm that it is of an acceptable scientific standard.
